# Case Report: Stent Retriever Thrombectomy of Acute Basilar Artery Occlusion *via* the Type 1 Proatlantal Intersegmental Artery

**DOI:** 10.3389/fneur.2022.812458

**Published:** 2022-05-23

**Authors:** Lei Zhao, Lijun Yang, Xiaosong Liu, Xiaoliang Wang, Gengshen Zhang, Jianliang Wu

**Affiliations:** Department of Neurosurgery, The Second Hospital of Hebei Medical University, Shijiazhuang, China

**Keywords:** proatlantal intersegmental artery, acute basilar artery occlusion, thrombectomy, stroke, case report

## Abstract

Stent retriever thrombectomy (SRT) is one of the most effective methods for the recanalization of acute basilar artery occlusion (ABAO). The proatlantal intersegmental artery (PIA) is a rare carotid-vertebrobasilar anastomosis. Recognition of this rare form of anastomosis is particularly important for the rapid establishment of positive blood flow in patients with ABAO. In this case, the patient had a rare, left type 1 PIA. The right vertebral artery (VA) was tenuous and did not enter the cranium. We performed a thrombectomy of the ABAO by inserting a catheter *via* the type 1 PIA. The complete recanalization of basilar artery (BA) flow was achieved following two stent retractions; however, the patient eventually died of brain stem hemorrhage.

## Introduction

Acute basilar artery occlusion (ABAO) is the most serious type of acute ischemic stroke (AIS). Failure to achieve rapid vascular recanalization can lead to high mortality and disability rates and a poor clinical prognosis. The safety and efficacy of stent retriever thrombectomy (SRT) in the treatment of acute large vessel occlusion (LVO) in the anterior circulation have been confirmed in many large clinical trials (1). In recent years, SRT has been attempted for the treatment of ABAO, which has been rigorously evaluated and has achieved good results (2).

The proatlantal intersegmental artery (PIA) is a rare carotid-vertebrobasilar anastomosis (CVA) that is often accompanied by vertebral artery (VA) and posterior communicating artery hypoplasia and is the main blood supply for the vertebrobasilar artery (3). In this case, the patient had a rare, left type 1 PIA. We did not perform a preoperative CT angiography (CTA) examination to assess vascular conditions, which led to some difficulties in identifying effective catheter access during the emergency thrombectomy.

## Case Description

A formerly healthy, 33-year-old man presented to our emergency department 6 h after the onset of unconsciousness. The patient had no history of hypertension, diabetes mellitus, or cardiovascular disease. Upon admission, neurological examination revealed a severe disturbance of consciousness with a Glasgow Scale (GCS) score of 4 (E1V1M2). The bilateral pupil size was about 1.5 mm, and light reflex was absent. When the patient was subjected to intense painful stimuli, the limbs were hyperextended. Bilateral pathological signs were not elicited. His initial National Institutes of Health Stroke Scale (NIHSS) scores were 39.

Non-contrast head CT demonstrated no significant infarction or cerebral hemorrhage. Due to problems with the picture archiving and communication system (PACS) in the hospital, CTA and MRI examinations were not performed. We were concerned that moving the patient to another hospital could delay optimal treatment. The combination of clinical symptoms and a physical examination of the nervous system led to a clinical diagnosis of AIS of the posterior circulation. He was taken for a thrombectomy.

**Figure 1:**
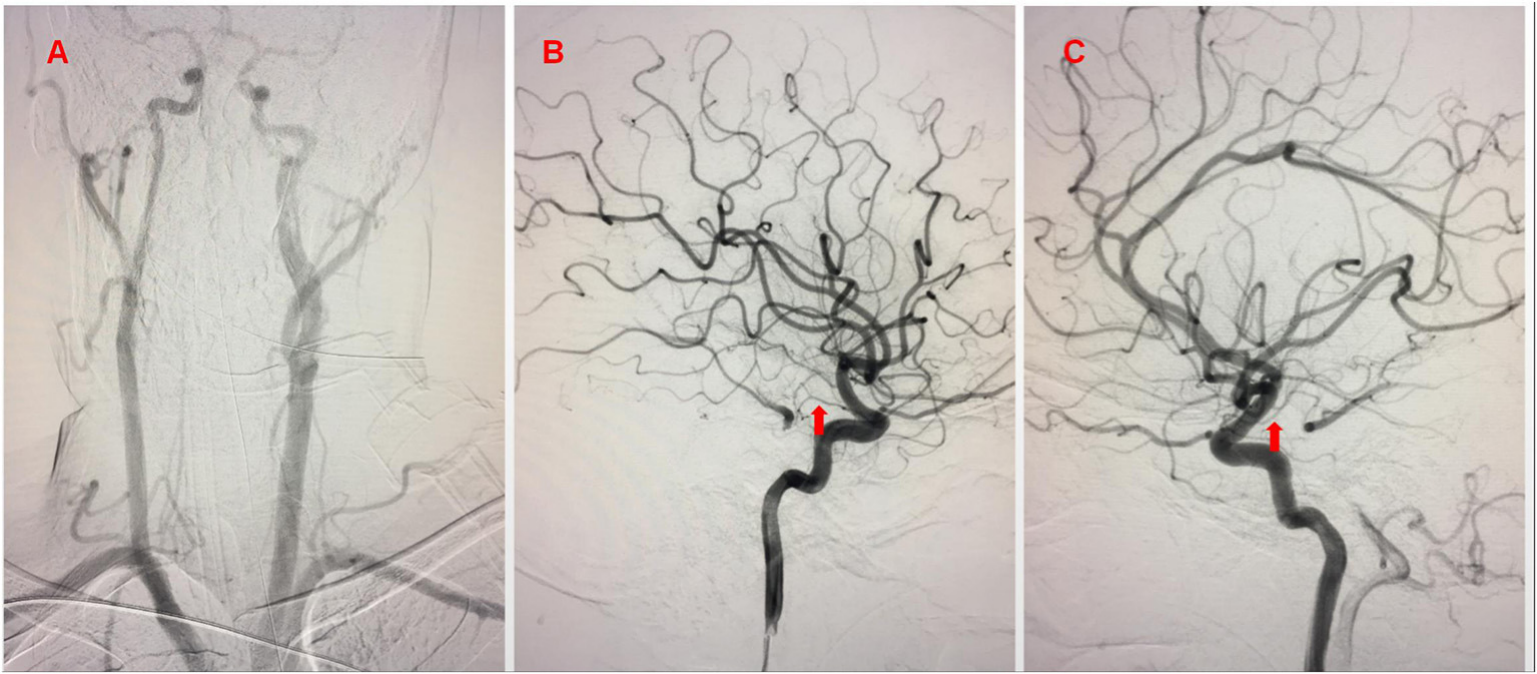
**(A)** Aortic arch catheter angiogram showing the right vertebral artery (VA) was tenuous and did not enter the skull; the left VA was absent. **(B,C)** Lateral internal carotid artery (ICA) catheter angiogram showed that bilateral posterior communication arteries were stunted (arrow) and collateral circulation was poor.

We performed emergency digital subtraction angiography (DSA). The aortic arch catheter angiogram showed that the patient's right VA was tenuous and did not enter the skull; the left VA was absent. The lateral internal carotid artery (ICA) catheter angiogram showed that the bilateral posterior communication arteries were stunted, and that collateral circulation was poor (Figure 1).

**Figure 2:**
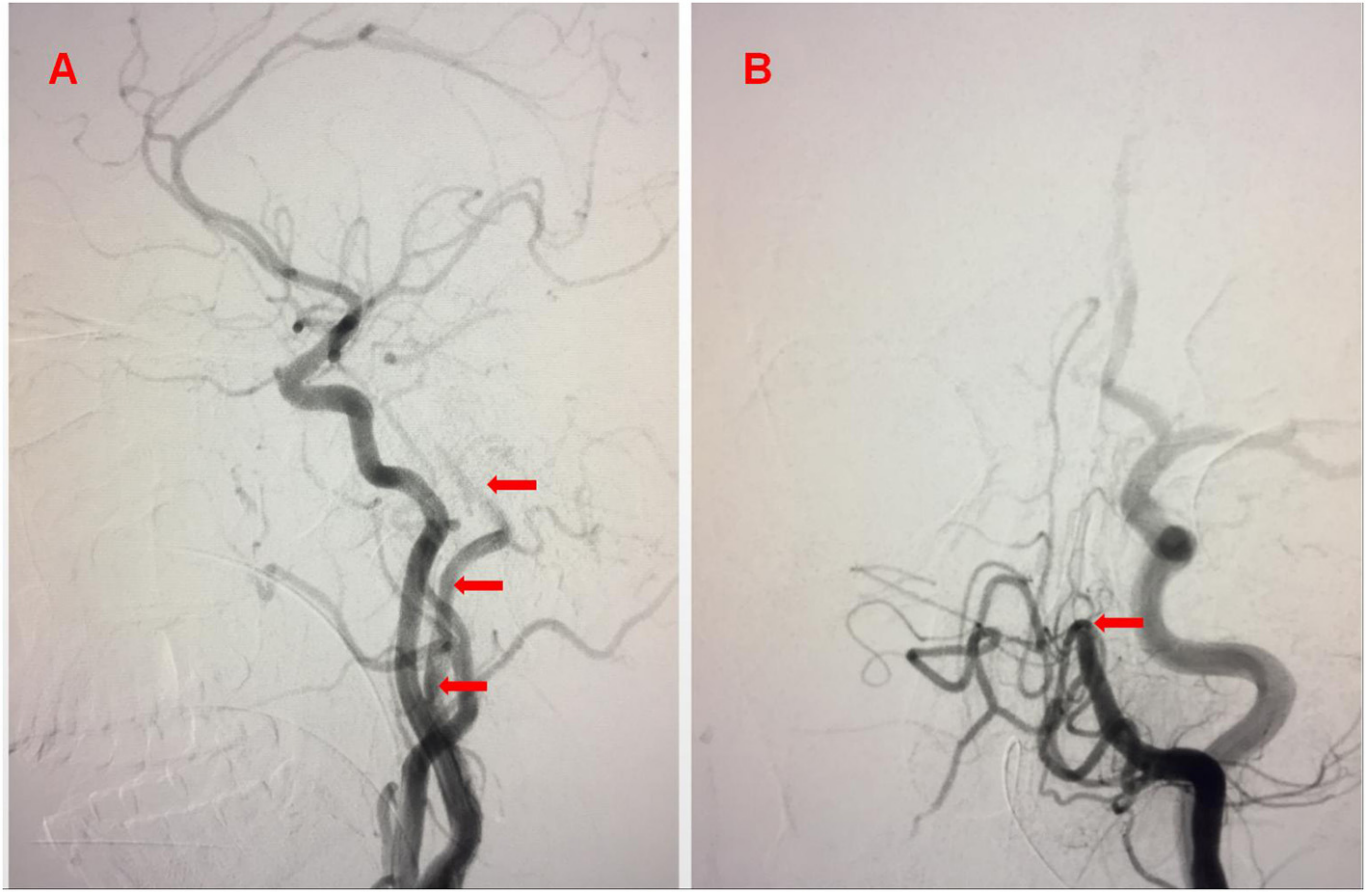
**(A)** Lateral ICA catheter angiogram showing a large collateral vessel [type1 proatlantal intersegmental artery (PIA)] was found emanating from the carotid segment of the ICA (arrow). **(B)** Angiography *via* type1 PIA demonstrated the basilar artery (BA) occluded far from the anterior inferior cerebellar artery (arrow).

During the left common carotid artery (CCA) angiography, a large collateral vessel (type 1 PIA) was found emanating from the carotid segment of the internal carotid artery (ICA). The angiographic catheter was placed at the beginning of this abnormal collateral vessel, and we saw that this vessel rose vertically and continued into the left vertebrobasilar artery. The BA was occluded far from the anterior inferior cerebellar artery (Figure 2).

## Treatment

**Figure 3:**
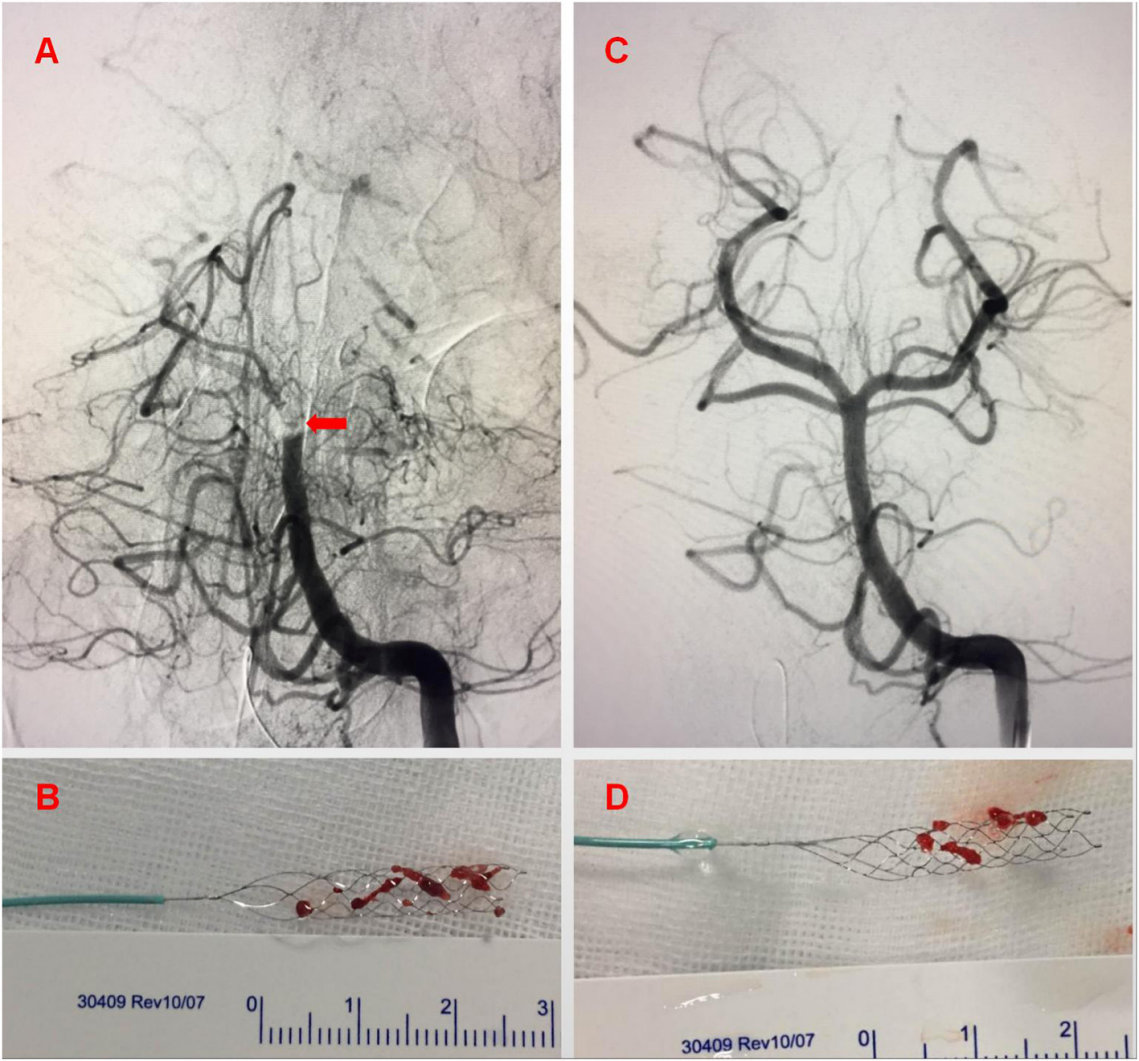
Some residual thrombus remained at the top of the basilar artery **(A)** after the first retrieval of the thrombus with stent **(B)** (arrow). After the second retrieval of the thrombus **(D)**, the basilar artery perfusion recovered completely **(C)**.

A 6 mm × 30 mm Solitaire stent was chosen for mechanical thrombectomy *via* the type 1 PIA. The complete recanalization of BA flow was achieved following two stent retractions, reaching thrombolysis in cerebral infarction (TICI) level 3 (Figure 3). Immediate post-operative head CT showed brain stem hemorrhage. The patient was returned to NICU with an oral trachea cannula. The systolic blood pressure was strictly controlled. Since the patient's autonomous respiration was weak and oxyhemoglobin saturation was difficult to maintain, we gave the patient assisted mechanical ventilation and other related treatments.

## Outcome and Follow-Up

The patient eventually died of a brain stem hemorrhage.

## Discussion

During the early development of the human embryo, there are various anastomotic channels between the carotid artery and the vertebrobasilar artery system (4). In most cases, CVAs usually regress during embryonic development. However, there are rare cases in which these original anastomoses do not disappear, but rather persist into adulthood. These are known as the persistent primitive PIA, the persistent hypoglossal artery (PHA), the persistent auricular artery (PAA), and the persistent trigeminal artery (PTA).

Proatlantal intersegmental artery is a rare CVA with an incidence of ~0.02% (5). The PIA is divided into two types according to two differing origins. Type 1 PIA originates from the dorsal cervical segment of the ICA at the level of the C2-3 vertebra of the cervical spine. Type 2 PIA originates from the initial part of the external carotid artery at the level of the C2-3 vertebra of the cervical spine. Both types of PIA travel upward and enter the skull through the foramen magnum. PIA is often associated with a transient ischemic attack (TIA), vertebrobasilar insufficiency (VBI), and even cerebral hemisphere or posterior circulation infarction (3).

Type I PIA and PHA are easily confused because they both originate from the extracranial segment of the ICA. The main differences between these two vessels are as follows: (1) PHA originates from the level of the C1 vertebra of the ICA or the C1-2 intervertebral space, which is higher than that of type I PIA; (2) PHA has a longer vertical lift than type 1 PIA; (3) type I PIA enters the cranium through the foramen magnum, while PHA enters the cranium through the sublingual neural tube (4). Park JS et al. recently reported a case of acute basilar artery occlusion treated with Endovascular thrombectomy *via* PHA (6). In our case, a primitive anastomotic artery had originated from the dorsal part of the cervical segment of the left ICA and the upper part of the mandibular angle (at the level of the C2-3 vertebral body); this had moved upward and entered the skull through the foramen magnum. Therefore, we speculated that the primitive anastomotic artery was a type 1 PIA (not confirmed by three-dimensional CTA).

Good collateral circulation and distal BAO are known to be independent predictors of clinical outcomes after intravascular treatment in patients with ABAO (7). Montechiari et al. reported a case of type 1 PIA associated with an ipsilateral embryonal posterior cerebral artery in 2013 (8). However, the patient's bilateral posterior communication arteries were stunted. Although the BA was completely recanalized by SRT and blood perfusion was at TICI level 3, the patient's poor collateral circulation may have resulted in a very poor prognosis even in the absence of a fatal brain stem hemorrhage.

## Conclusion

Proatlantal intersegmental artery itself does not require treatment; however, because of its special anatomy, the vertebrobasilar system must share blood flow with the carotid system. Thus, PIA may be associated with a higher incidence of posterior circulation ischemic events. Cranio-carotid CTA examination is particularly important for ABAO, especially when ABAO is combined with PIA and other rare vascular variants.

## Data Availability Statement

The original contributions presented in the study are included in the article/supplementary material, further inquiries can be directed to the corresponding author/s.

## Ethics Statement

Written informed consent was obtained from the individual(s) and/or minor(s)' legal guardian/next of kin for the publication of any potentially identifiable images or data included in this article.

## Author Contributions

LZ, LY, XL, XW, GZ, and JW contributed to the conception and design, acquisition and interpretation of the case data, drafting and revising of the article critically for important intellectual content, final approval of the version published, and agreement to be accountable for the accuracy or integrity of the article. All authors contributed to the article and approved the submitted version.

## Conflict of Interest

The authors declare that the research was conducted in the absence of any commercial or financial relationships that could be construed as a potential conflict of interest.

## Publisher's Note

All claims expressed in this article are solely those of the authors and do not necessarily represent those of their affiliated organizations, or those of the publisher, the editors and the reviewers. Any product that may be evaluated in this article, or claim that may be made by its manufacturer, is not guaranteed or endorsed by the publisher.
